# Validation of the IDF-DAR risk assessment tool for Ramadan fasting in patients with diabetes in primary care

**DOI:** 10.3389/fcdhc.2025.1426120

**Published:** 2025-03-21

**Authors:** Latifa Baynouna Alketbi, Bachar Afandi, Nico Nagelkerke, Hanan Abdubaqi, Ruqaya Abdulla Al Nuaimi, Mariam Rashed Al Saedi, Fatima Ibrahim Al Blooshi, Noura Salem Al Blooshi, Aysha Mohammed AlAryani, Nouf Mohammed Al Marzooqi, Amal Abdullah Al Khouri, Shamsa Ahmed Al Mansoori, Mohammad Hassanein

**Affiliations:** ^1^ Academic Affairs Department, Ambulatory Healthcare Services, Al Ain, United Arab Emirates; ^2^ Internal Medicine Department, Tawam Hospital, Al Ain, United Arab Emirates; ^3^ College of Medicine, United Arab Emirates University, Al Ain, United Arab Emirates; ^4^ Internal Medicine Department, Dubai Hospital, Dubai, United Arab Emirates

**Keywords:** diabetes mellitus, Ramadan, fasting, adverse events, risk assessment

## Abstract

**Introduction:**

In patients with diabetes intending to fast, Ramadan, risk assessment, and stratification are essential for an individualized treatment plan. It seems that the new IDF-DAR risk stratification tool (International Diabetes Federation - Diabetes and Ramadan Alliance) has become the primary tool in this setting. This study aims to validate this tool in the Abu Dhabi population.

**Method:**

The assessment was performed before Ramadan, followed by an evaluation of any significant outcome after Ramadan through tele-interview and an electronic medical records review. Patients were included if the attending physicians used the tool in the risk assessment of the patients within 6 weeks before Ramadan 1,444 (CE 2022) in the AHS healthcare center.

**Results:**

The study included 435 patients. Half (51.7%) were in the low-risk category of the IDF-DAR risk stratification tool, 28.5% were in the moderate-risk category, and 19.8% were in the higher-risk category. Of the total patients, 81.3% fasted during the entire Ramadan period and 18.7% attempted to fast. A total of 14 (3.8%) patients were admitted at least once, and 56 (12.9%) had at least one significant event, including admission to the hospital. Using univariable logistic regression, the occurrence of adverse events was significantly associated with more days not fasted, *B* = −0.126, *p* < 0.001, OR = 0.88 (0.839–0.927). Using multivariable logistic regression, and after controlling for all variables studied, other risk factors identified with the occurrence of adverse events in this study were as follows: being in the low-risk category of the DAR risk assessment tool, *B* = −1.1, OR = 0.34 (0.157–0.744), *p* = 0.0072; being in the frail category compared to the reference category, the robust category, *B* = 1.54, OR = 4.6 (1.3–16.6), *p* = 0.018; and older age *B* = −0.034, OR = 0.966 (0.938–0.995). There was no significant difference between moderate- and high-risk categories in the occurrence of significant adverse events (SAEs). Similar determinants of fasting were identified during the entire Ramadan period using multivariable logistic regression.

**Conclusion:**

According to the IDF-DAR risk assessment, patients with diabetes in the low-risk category had a better outcome than those in the moderate- or high-risk categories regarding SAEs. Another independent risk factor is if the patient is frail, according to the FRAIL scoring.

## Background

The health effects of changes in the diet and lifestyle of patients with diabetes during Ramadan are of increasing interest in terms of estimating the possible risks and benefits to better inform management guidelines, including recommending exemptions from fasting and optimal medication adjustments. For the whole month of Ramadan, Muslims must abstain from eating and drinking from dawn to sunset, and these fasting hours are accompanied by a change in sleep and primary meal times. Accumulating evidence, including a recent systematic review, confirms that the incidences of complications during Ramadan are minimally higher than at other times of the year in high-risk patients with diabetes (1). However, the ability of patients’ bodies to adjust to these changes is variable, and many factors, such as comorbidities and diabetic complications, may influence it. Therefore, many international societies have issued guidelines to guide physicians and patients to fast Ramadan safely (2).

In patients with diabetes, risk assessment and stratification are essential for an individualized treatment plan. It seems that the new IDF-DAR risk stratification tool (International Diabetes Federation - Diabetes and Ramadan Alliance) has become the primary tool in this setting. It was developed based on the available evidence and the consensus of experts who included factors thought or found to increase the risk of adverse events in patients with diabetes. A score is generated as collective points given to the different factors if these risk factors were present (2). It was found to be valid in predicting both the ability to fast during Ramadan and the likelihood of getting hypoglycemia, hyperglycemia, or significant adverse events (SAEs) (3–9). Validating this calculator in different settings will facilitate more perception and the applicability of the tool.

The Abu Dhabi Emirates context represents a high-resource Muslim country with a high prevalence of diabetes (2). Providing appropriate, effective, quality care for this large population in preparation for Ramadan fasting is a priority for better quality of life and less risk. Globally, fasting was found to be efficient for the management of diabetes (10). Assessment tools that can assist in stratifying patients with diabetes are critical in avoiding stressing high-risk patients.

For a few years, Ambulatory Healthcare Services had an initiative to counsel and adjust the care plan for patients with chronic diseases who intend to fast during Ramadan. The IDF-DAR practical guidelines, including the IDF-DAR risk stratification tool published in 2021, were used to assess risk and assist in decision-making for people with diabetes. The tool was built into the electronic medical records (EMR) system. Physicians were encouraged to use it to help stratify patients with diabetes and guide their counseling on decisions regarding fasting. This study aimed to validate the IDF-DAR risk stratification tool by comparing the patients’ outcomes after Ramadan to the assessed baseline, the IDF-DAR risk stratification score before Ramadan. This outcome-based risk stratification is important for guiding clinical patient care decisions.

## Method

Ambulatory Healthcare Services has structured chronic disease clinics. Within these clinics, an annual initiative targeted patients with chronic disease to counsel them and adjust their care plan in preparation for fasting during Ramadan if medically appropriate. Integrating Ramadan fasting-related counseling in chronic disease patient visits is preceded by an educational event targeting physicians on new updates in this area. The IDF-DAR practical guidelines were included in the educational event, including the IDF-DAR risk stratification tool published in 2021. The AHS team requested to use the tool within the EMR system, which was built internally within CERNER EMR. The training was conducted, and physicians were encouraged to use it to help stratify patients with diabetes and guide their counseling on decisions regarding fasting.

### Data collection

This is a prospective observational study. Assessments were performed for all participants at two time points: 6 weeks before Ramadan 1,444 (CE 2022) in the AHS healthcare center and again after Ramadan. Patients were included if the attending physicians used the IDF-DAR risk stratification tool as a pre-Ramadan assessment. As per the IDF-DAR practical guidelines, all patients with diabetes who intend to fast during Ramadan should be screened using this tool. An EMR report was extracted before Ramadan. All patients in the EMR report were contacted and asked for consent, and an assessment of frailty using the FRAIL scale was carried out. Besides the IDF-DAR risk assessment and FRAIL score, no other clinical data were collected except those routinely ordered before Ramadan. Therefore, patients from the AHS centers assessed as part of their routine visits were included.

After Ramadan, family medicine residents collected data through tele-interview. They called the patients, and if they consented to participate, data were collected regarding fasting and significant history during Ramadan. As Abu Dhabi has integrated electronic health records, data collected through patient tele-interviews could be validated through the EMR chart review. Patient privacy was maintained during tele-interviews as it was done from the AHS centers, and consent for the interview and EMR review was taken.

Review of EMR was done after Ramadan. Important demographics and medical history not included in the IDF-DAR risk assessment were collected in addition to clinical data such as laboratory results within 3 months before Ramadan and 3 months after Ramadan that were determined as part of the patient’s routine care. These laboratory results included HBA1C, renal function, systolic blood pressure (SBP), diastolic blood pressure (DBP), and body mass index (BMI). Frailty assessment was performed for patients 60 years or older, and the FRAIL tool was used. It is a validated tool with five questions demonstrating strong evidence for predicting clinical outcomes (11–14). The five questions were related to fatigue; resistance or climbing stairs; ambulation, i.e., walking a couple of blocks; number of chronic illnesses; and losing weight by more than 5%.

Outcome assessment after Ramadan included fasting status, significant health events, and time of admission into care collected after Ramadan. Only events occurring during Ramadan were considered in this study and for those who did fast after their fast. Surveillance started on the first day of Ramadan and continued until the end of the holy month. Events included unplanned admissions, a history of hypoglycemia, and significant symptoms that required breaking fasting, such as dizziness, fainting, and fever.

### Data analysis

**Figure 1:**
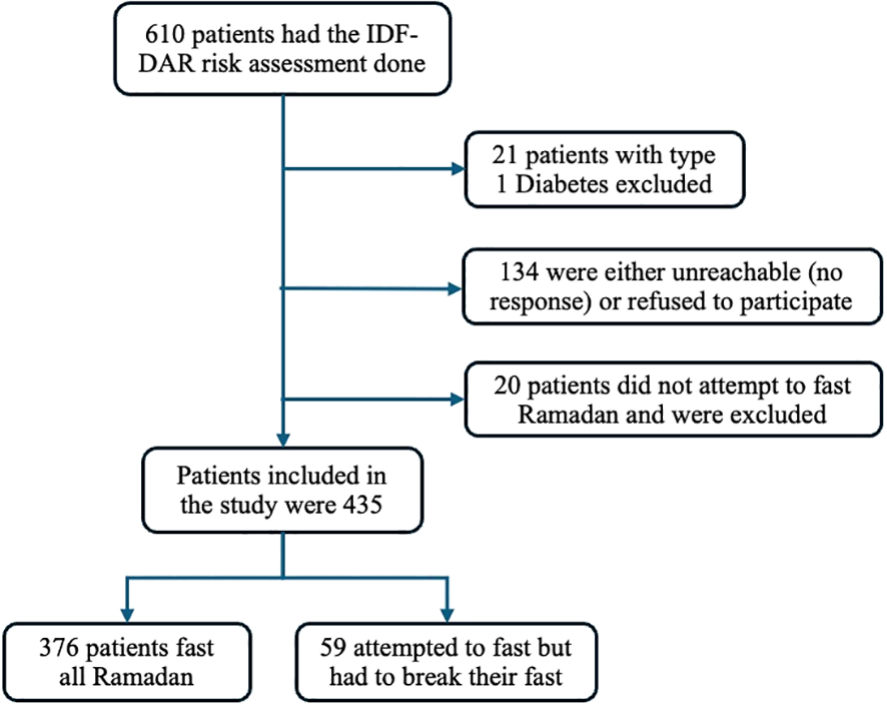
Flow diagram for the study sample with excluded and included subjects.

Out of the 610 patients with diabetes who had the IDF-DAR risk assessment score calculated, 435 were included in the analysis (**Figure 1**). Twenty patients who did not attempt to fast for a single day were excluded. Patients with type 1 diabetes were also excluded due to their small number in primary care (21 patients in total), which can result in a heterogeneous sample. There were 134 patients with no response or who refused to participate.

Data analysis was performed using SPSS v27. Frequencies, cross-tabulation, and regression analysis were used. Logistic regression was used to test the dependent variable outcomes studied, significant event occurrence, and hypoglycemia, with all variables collected and included to test possible significant associations. Hosmer and Lemeshow chi-square was used to test for goodness of fit and calibration of logistic regression models.

## Results

According to the IDF-DAR Assessment tool, half of the study participants (51.7%) were in the low-risk category, of which 89.8% fasted during Ramadan. Among the study participants, 28.5% were categorized as moderate risk and 84.7% fasted during Ramadan; 19.8% were in the higher-risk category, and those who fasted during Ramadan were 80.2%.

**Table 1** shows the patients’ demographics, with almost two-thirds of the patients belonging to the 50 years or above age group and 16.1% belonging to the above 70 age group. Those between 40 and 50 comprised 18.4%. Older patients were in the higher-risk category per the DAR stratification. For example, nearly one-quarter of them were in the higher-risk group in the age groups above 50 years compared to 2.3% and 11.6% in the age groups 31 to 40 years and 41 to 50 years, respectively. Male patients comprised 43.4% of the sample, and UAE nationals comprised the majority (74.5%). Of the included patients, 57% had diabetes for more than 10 years. Diabetes control became progressively worse as the DAR risk category increased, with an average HbA1C of 7.18, 8.2, and 9 for the low-, moderate-, and high-risk categories, respectively. **Table 1** shows the distribution of comorbidities and the medications used by patients with diabetes.

**Table 1:** Study subjects’ characteristics.

		Low Risk	Moderate Risk	High Risk	All
Age groups	≤30	2	1	0	3
		0.90%	0.80%	0.00%	0.70%
	31–40	16	8	2	26
		7.10%	6.50%	2.30%	6.00%
	41–50	49	21	10	80
		21.80%	16.90%	11.60%	18.40%
	51–60	74	30	22	126
		32.90%	24.20%	25.60%	29.00%
	61–70	60	47	23	130
		26.70%	37.90%	26.70%	29.90%
	>70	24	17	29	70
		10.70%	13.70%	33.70%	16.10%
Gender	Female	130	64	52	246
		57.80%	51.60%	60.50%	56.60%
	Male	95	60	34	189
		42.20%	48.40%	39.50%	43.40%
Nationality	Non-UAE	53	29	29	111
		23.60%	23.40%	33.70%	25.50%
	UAE	172	95	57	324
		76.40%	76.60%	66.30%	74.50%
Duration of diabetes (years)	A duration of <10	140	35	12	187
		62.20%	28.20%	14.00%	43.00%
	A duration of ≥10	85	89	74	248
		37.80%	71.80%	86.00%	57.00%
Renal complications/comorbidities	eGFR < 30 mL/min	0	0	4	4
		0.00%	0.00%	4.70%	0.90%
	eGFR >60 mL/min	222	110	56	388
		98.70%	88.70%	65.10%	89.20%
	eGFR 30–45 mL/min	0	3	15	18
		0.00%	2.40%	17.40%	4.10%
	eGFR 45–60 mL/min	3	11	11	25
		1.30%	8.90%	12.80%	5.70%
MVD complications/comorbidities		1	1	0	2
		0.40%	0.80%	0.00%	0.50%
	No MVD	213	92	38	343
		94.70%	74.20%	44.20%	78.90%
	Stable MVD	11	31	40	82
		4.90%	25.00%	46.50%	18.90%
	Unstable MVD	0	0	8	8
		0.00%	0.00%	9.30%	1.80%
Presence of hypoglycemia	Hypoglycemia less than 1	13	8	19	40
		5.80%	6.50%	22.10%	9.20%
	Hypoglycemia unawareness	0	1	3	4
		0.00%	0.80%	3.50%	0.90%
	Multiple weekly hypoglycemia	0	1	6	7
		0.00%	0.80%	7.00%	1.60%
	No hypoglycemia	212	114	57	383
		94.20%	91.90%	66.30%	88.00%
	Recent severe hypoglycemia	0	0	1	1
		0.00%	0.00%	1.20%	0.20%
Pregnancy	No	142	72	56	270
		100.00%	97.30%	98.20%	98.90%
	Yes	0	2	1	3
		0.00%	2.70%	1.80%	1.10%
Frailty and cognitive function as assessed by physicians	>70 years old with no home support	0	5	10	15
		0.00%	4.00%	11.60%	3.40%
	Impaired cognitive function or frail	0	0	10	10
		0.00%	0.00%	11.60%	2.30%
	No frailty or loss in cognitive function	224	118	66	408
		99.60%	95.20%	76.70%	93.80%
HbA1C	<7.5	144.00	38.00	20.00	202.00
		72.00	34.00	26.00	57.00
	7.5–9	41.00	44.00	25.00	110.00
		20.00	0.39	0.33	28.00
	>9	16.00	31.00	32.00	79.00
		8.00	27.00	42.00	20.00
Physical labor		0	1	0	1
	0.00%	0.80%	0.00%	0.20%
	Moderate to intense pain	14	24	12	50
		6.20%	19.40%	14.00%	11.50%
	No physical labor	211	99	74	384
		93.80%	79.80%	86.00%	88.30%
Self-monitoring of blood glucose	Conducted as indicated	162	62	32	256
		72.00%	50.00%	37.20%	58.90%
	Indicated but conducted sub-opt	53	46	32	131
		23.60%	37.10%	37.20%	30.10%
	Indicated but not conducted	9	16	22	47
		4.00%	12.90%	25.60%	10.80%
Fasting hours	<16 h	206	108	74	388
		91.60%	87.10%	86.00%	89.20%
	≥16 h	19	16	12	47
		8.40%	12.90%	14.00%	10.80%
Ischemic heart diseases	No IHD	218	110	67	395
		97.80%	90.20%	78.80%	91.90%
	IHD	5	12	18	35
		2.20%	9.80%	21.20%	8.10%
Stroke	No stroke	222	121	82	425
		99.60%	98.40%	96.50%	98.60%
	Stroke	1	2	3	6
		0.40%	1.60%	3.50%	1.40%
Hypertension	No hypertension	96	45	20	161
		43.60%	36.60%	23.80%	37.70%
	Hypertension	124	78	64	266
		56.40%	63.40%	76.20%	62.30%
GLP-1 receptor agonist	Not on GLP-1 receptor agonist	117	50	26	193
		52.00%	40.30%	30.20%	44.40%
	On GLP-1 receptor agonist	108	74	60	242
		48.00%	59.70%	69.80%	55.60%
DPP-4 inhibitor	Not on DPP-4 inhibitor	74	26	17	117
		32.90%	21.00%	19.80%	26.90%
	On DPP-4 inhibitor	151	98	69	318
		67.10%	79.00%	80.20%	73.10%
SGLT2 inhibitor	Not on SGLT2 inhibitor	96	38	17	151
		42.70%	30.60%	19.80%	34.70%
	On SGLT2 inhibitor	129	86	69	284
		57.30%	69.40%	80.20%	65.30%
Sulfonylurea	Not on sulfonylurea	95	33	18	146
		42.20%	26.60%	20.90%	33.60%
	On sulfonylurea	130	91	68	289
		57.80%	73.40%	79.10%	66.40%
Insulin	Not on insulin	125	48	11	184
		55.60%	38.70%	12.80%	42.30%
	On insulin	100	76	75	251
		44.40%	61.30%	87.20%	57.70%
TZDs	Not on TZDs	122	54	29	205
		54.20%	43.50%	33.70%	47.10%
	On TZDs	103	70	57	230
		45.80%	56.50%	66.30%	52.90%
CKD	No CKD	221	109	62	392
		98.20%	87.90%	72.10%	90.10%
	CKD	4	15	24	43
		1.80%	12.10%	27.90%	9.90%
Days not fasted coded	Did fast all days	202	105	69	376
		89.80%	84.70%	80.20%	86.40%
	−1 to −4	10	10	7	27
		4.40%	8.10%	8.10%	6.20%
	−5 to −9	5	3	3	11
		2.20%	2.40%	3.50%	2.50%
	−10 to −14	2	1	1	4
		0.90%	0.80%	1.20%	0.90%
	−15 to −19	2	3	3	8
		0.90%	2.40%	3.50%	1.80%
	−20 to −24	1	0	1	2
		0.40%	0.00%	1.20%	0.50%
	−25 to −30	3	2	2	7
		1.30%	1.60%	2.30%	1.60%
Admitted to hospital	No	184	107	66	357
		98.90%	97.30%	88.00%	96.20%
	Yes	2	3	9	14
		1.10%	2.70%	12.00%	3.80%
Significant event or admitted to hospital	No	207	103	69	379
		92.00%	83.10%	80.20%	87.10%
	Yes	18	21	17	56
		8.00%	16.90%	19.80%	12.90%
Frail cat	Robust	47	29	19	95
		52.80%	45.30%	36.50%	46.30%
	Prefrail	38	33	19	90
		42.70%	51.60%	36.50%	43.90%
	Frail	4	2	14	20
		4.50%	3.10%	26.90%	9.80%
Total		225	124	86	435
		51.7%	28.5%	19.8%	100.00%

Regarding outcome, from the entire sample, 14 (3.8%) were admitted at least once and 56 (12.9%) had at least one significant event, including hospital admission. This was progressive among the three risk groups as per the DAR tool with 2 (1.1%), 3 (2.7%), and 9 (12%) admissions in the low-, moderate-, and high-risk groups, respectively, and 18 (8%), 21 (16.9%), and 17 (19.8%) among low-, moderate-, and high-risk groups, respectively. Those who did not fast had an event rate of 8 (42.1%) compared to 56 (12.9%) among those who fasted or attempted to fast.

**Figure 2:**
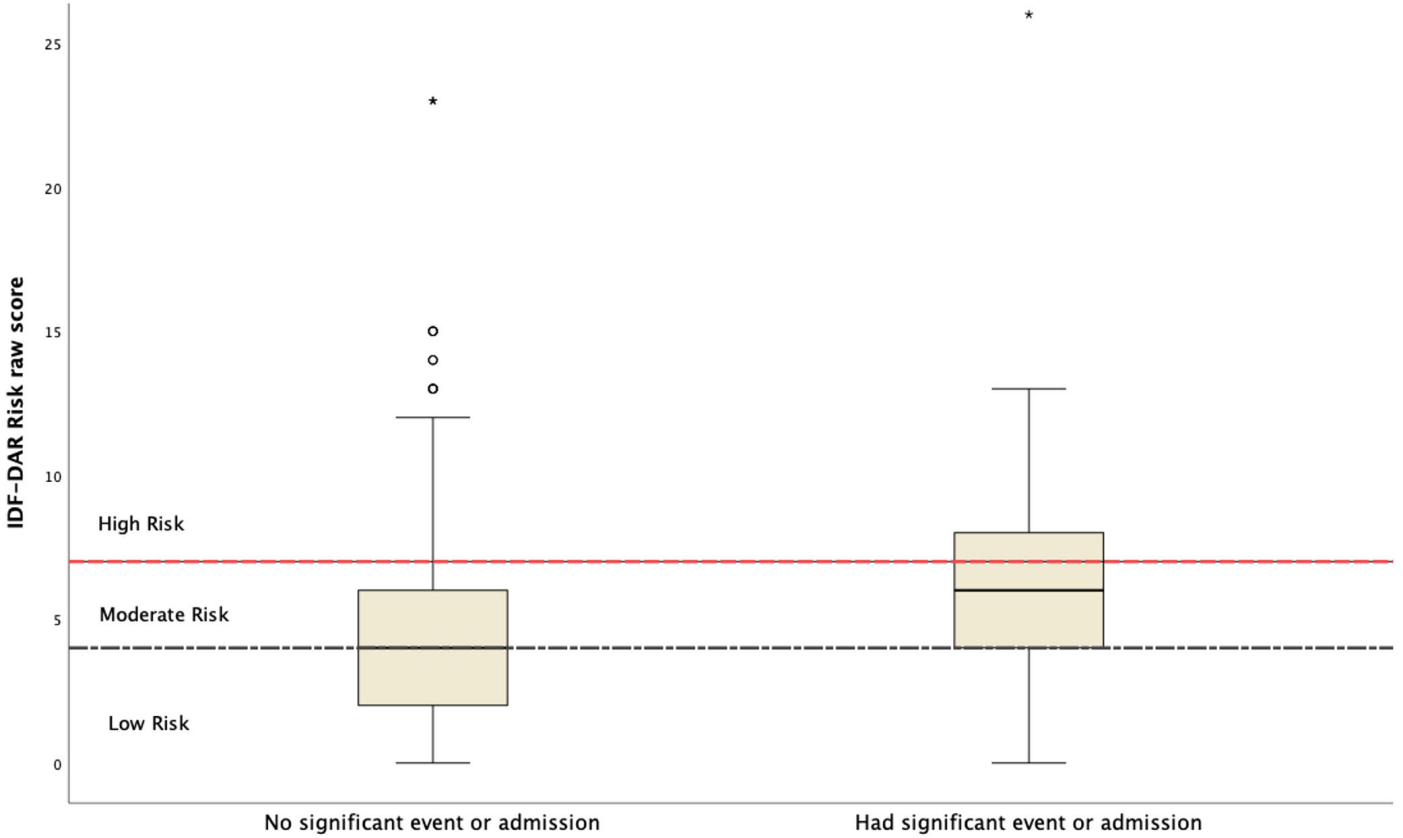
The IDF-DAR raw risk score in relation to the occurrence of significant adverse events (reported significant symptom or hospital admission).

Using univariable logistic regression, the occurrence of adverse events was significantly associated with more days not fasted, *B* = −0.126, *p* < 0.001, OR = 0.88 (0.839–0.927). Using multivariable logistic regression, and after controlling for all variables studied, other risk factors identified with the occurrence of adverse events in this study were as follows: being in the low-risk category of the DAR risk assessment tool, *B* = −1.1, OR = 0.34 (0.157–0.744), *p* = 0.0072; being in the frail category compared to the reference category, the robust category, *B* = 1.54, OR = 4.6 (1.3–16.6), *p* = 0.018; and older age, *B* = −0.034, OR = 0.966 (0.938–0.995). Interestingly, there was no significant difference between moderate- and high-risk categories in the occurrence of SAE in **Table 2A** and **Figure 2**. Hosmer and Lemeshow chi-square = 3.36, *p* = 0.91.

Table 2Predictors of (A) significant adverse events occurring during Ramadan and (B) fasting during Ramadan among the whole cohort of 435 participants.

**Table:** 

Predictors of significant adverse events (SAE) during Ramadan.					
	*B*	*p*-value	OR	95% CI for OR (*B*)	
Age	−0.034	0.023	0.966	0.938	0.995
Robust frailty category (Reference)					
Prefrail	0.170	0.692	1.185	0.511	2.747
Frail	1.537	0.018	4.649	1.305	16.558
High-risk IDF-DAR risk category (Reference)					
Low-risk IDF-DAR risk category	−1.074	0.007	0.342	0.157	0.744
Moderate-risk IDF-DAR risk category	−0.124	0.749	0.883	0.412	1.893

Logistic regression

**Table:** 

Predictors of fasting during Ramadan					
	*B*	*p*-value	OR	95% CI for OR (*B*)	
AGE	0.026	0.076	1.026	0.997	1.056
Robust frailty category (Reference)					
Prefrail frailty category	−0.166	0.686	0.847	0.380	1.889
Frail frailty category	−1.340	0.034	0.262	0.076	0.904
High-risk IDF-DAR risk category (Reference)					
Low-risk IDF-DAR risk category	0.758	0.045	2.134	1.017	4.478
Moderate-risk IDF-DAR risk category	0.230	0.558	1.259	0.583	2.715

Logistic regression.

Similar determinants of fasting during Ramadan were identified using multivariable logistic regression (**Table 2B**). Being in the lower-risk category of IDF-DAR doubles the possibility of fasting during Ramadan, *B* = 0.76, OR = 2.1 (1.01–4.5), compared to the high-risk group, and there is no difference between the moderate- and high-risk groups in completing fasting during Ramadan. Similar to the occurrence of ASE, being frail was a significant risk factor for not fasting during the entire Ramadan period, while older age was marginally significant, *p* = 0.076. Hosmer and Lemeshow chi-square = 8.597, *p* = 0.377.

The performance of the developed model from the logistic regression analysis is described in **Table 3**.

**Table 3:** Performance of the IDF-DAR tool and the model developed and from the logistic regression in predicting (A) SAE and (B) fasting during Ramadan.

A. Predicting SAE						
	Cut-off	Sensitivity	Specificity	*c* statistics	CI	
IDF-DAR score	3.5	67.3	54.4	0.610	0.527	0.692
Predicted probability	13.8	60.7	67.8	0.676	0.603	0.748

B. Prediction of fasting during Ramadan						
	Cutoff	Sensitivity	Specificity	*c* statistics	CI	
IDF-DAR score	7.5	27.6	87.2	0.581	0.495	0.667
Predicted Probability	86.7	60.4	67.7	0.623	0.541	0.705

**Figure 3:**
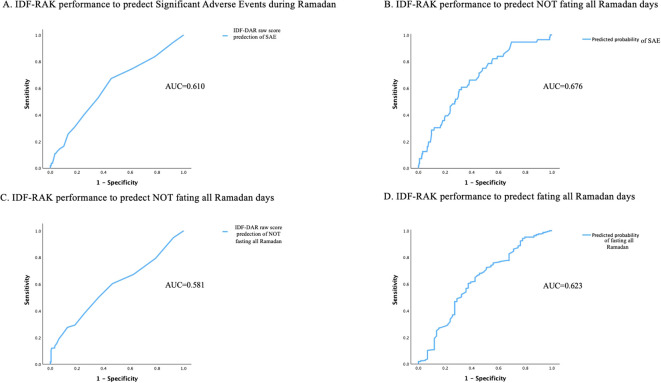
The IDF DAR raw risk score in relation to the occurrence of significant adverse events. (reported significant symptom or hospital admission). **(A)** IDF-RAK performance to predict Significant Adverse Events during Ramadan. **(B)** IDF-RAK performance to predict NOT fasting all Ramadan days. **(C)** IDF-RAK performance to predict NOT fasting all Ramadan days. **(D)** IDF-RAK performance to predict fasting all Ramadan days. AUC, Area Under the Curve; SAE, Significant Adverse Events.

The *c* statistics of the performance of the developed logistic regression model was better, 0.676, than the IDF-DAR alone, with 0.61 to predict outcomes studied. In addition, the sensitivity of the IDF-DAR alone was better in predicting SAE than a prediction of not fasting during the entire Ramadan period, 67.3% compared to 27.6%. The developed model, which included the FRAIL score, age, and the IDF-DAR score, performed better in detecting both studied outcomes. Sensitivity and specificity were similar, approximately 60% and 67%, respectively (**Figure 3**).

**Figure 4:**
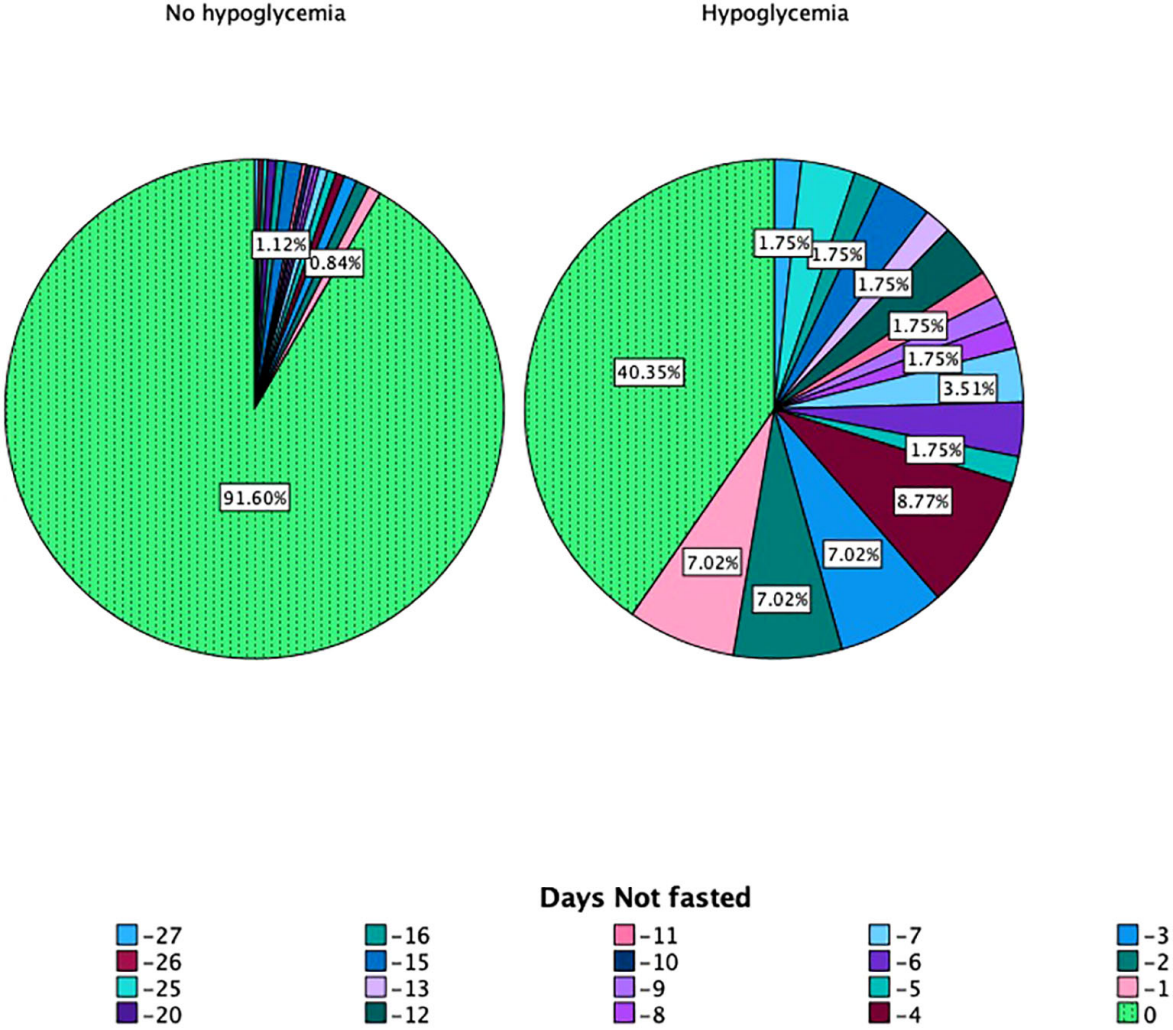
The incidence of hypoglycemia in relation to days fasted during Ramadan.

Another important outcome is the incidence of hypoglycemia, with 46 patients reporting hypoglycemic episodes during the month of Ramadan (11.7%). The high-risk group, as per the DAR stratification tool, had 10 patients experiencing these episodes during Ramadan (12.7% of the high-risk patients); the moderate-risk group had 18 episodes (15.8%), and the low-risk group had 18 episodes (9%) (**Table 4**). These hypoglycemia episodes were more likely among those who needed to break their fast, which was the only significant determinant of hypoglycemia as per logistic regression, *B* = −0.11, CI (0.85–0.94), SE = 0.027, *p* < 0.001 (**Figure 4**).

**Table 4:** Hypoglycemia incidence distributed by the IDF risk assessment score.

	Low Risk	Moderate Risk	High Risk	Total
No hypoglycemia	183	96	69	348
	91.00%	84.20%	87.30%	88.30%
Hypoglycemia	18	18	10	46
	9.00%	15.80%	12.70%	11.70%
Total	201	114	79	394
	100.00%	100.00%	100.00%	100.00%

## Discussion

Patients in the low-risk category, as per the DAR risk assessment tool, who fasted during Ramadan or attempted to fast had a significantly better outcome than those in the moderate- or high-risk categories during Ramadan regarding the occurrence of SAEs. They were at 70% less risk of developing an adverse event than those from the moderate- or high-risk categories. Those in the moderate- and high-risk categories were nearly close to each other in predicting patient outcomes during fasting. Although there was a noticeable increase in the risk of adverse events with increasing IDF-DAR risk category scores from low to moderate to high, as seen from the logistic regression controlling for other factors, it is suggested that counseling be provided for patients in the moderate- and high-risk groups equally.

This study reported more adverse events than a similar study in the same period in a diabetes center in Abu Dhabi-AlAin (3). Mohammad et al.’s study reported outcomes related to mainly hypoglycemic and hyperglycemic episodes. Only one patient in their cohort had been admitted. Therefore, this study adds an outcome that matters: fewer hospitalizations and incidence of illness in the lower-risk group. This observation was higher in this study than in Mohammad et al.’s. Surprisingly, their study had more hypoglycemic episodes than this study, 15% compared to 9.6%, respectively. However, other variables, such as diabetes control, insulin use, or diabetes duration, did not differ much between the two cohorts in explaining this difference. Such differences between cohorts warrant an investigation of the possible factors influencing outcomes. For example, this study setting is a primary care center, while the Mohammad et al. study is based in a diabetes-specialized center. There are potential cultural and healthcare system differences between the two settings that could explain such differences. Patients choosing to be followed in a specialized center could represent a different socioeconomic status or disease risk category. Moreover, differences in practice resources and medication use can influence such outcomes.

The risk of hypoglycemia may be related to other factors, such as the healthcare setting, health literacy, and self-management, but it is not associated with the patient’s risk profile. In another similar study to validate the DAR risk assessment tool in Bangladesh, Kamrul-Hasan et al. found that hypoglycemia and hyperglycemia risks were 3.74-fold and 3.86-fold higher in the high-risk group than in the low-risk group (5). Similar to this study, the episodes of hypoglycemia were lower than those of Mohammed et al.

This study supports Mohammad et al.’s and Kamrul-Hasan et al.’s study in that the DAR risk assessment tool helps stratify the risk of patients with diabetes intending to fast during Ramadan. Two additional independent risk factors were identified: previous fasting experience and patients’ frailty. The more days the patients did not fast, the more adverse events there were, which may indicate that they could not complete fasting due to poor health, and those who completed fasting are in a better health status. This highlights the importance of patients’ previous fasting experience, validating the DAR risk assessment tool question.

Frailty was found to be another independent risk factor in this study. No study used the FRAIL score to assess the outcome of fasting during Ramadan; therefore, this is an important addition discussed in another part of this study (15). This highlights the importance of studying new risk factors for adverse events, such as social factors, healthcare literacy, income level, and access to medical care. In the United States, for example, integrating social context into healthcare delivery has become a priority strategy due to accumulating evidence of it being determinantal in the outcome of patients with diabetes (16–18). The main limitation of this study is the possible variation among physicians who performed the clinical judgment and utilized the risk estimation calculator. This was found to be true in a study by Afandi et al., where classifying the risk of patients with diabetes during Ramadan fasting was found to be widely varied (19). They used case scenarios presented in a survey distributed to physicians and asked to evaluate patients’ risk utilizing the same DAR calculator. The variation was particularly higher in moderate-risk cases. Investigators attributed this to factors other than clinical judgment, such as personal, spiritual, and social factors. They recommended that risk categorization be customized to empower physicians to stratify each patient. Nevertheless, these factors, although important, in support of this risk assessment tool is the significant prediction of key outcomes that support the physicians’ judgment.

It is empirically recommended that validating this tool in different cultures and geographical regions will strengthen the prediction of outcomes. Important factors, such as healthcare literacy, self-management, and socioeconomic status, could significantly influence a patient’s ability to fast and manage diabetes. Future studies need to control for these social determinants of health.

Variations in individual physicians’ practice may influence the outcome, and it is also a limitation and possibility for future research. The medication change and review were not addressed during data collection. However, it was one of the aims of the counseling visits before Ramadan as part of routine care in preparation for Ramadan.

Another limitation could be the lack of blinding of patients and healthcare providers of the assessment result and subsequent counseling and change in management effect on outcome. Unfortunately, this limitation in assessing the risk of Ramadan fasting may not be possible to overcome; since the IDF-DAR tool was introduced a year before the study, it became a required assessment during the routine care of patients with diabetes before Ramadan. Depriving patients from knowing the risk and having physicians target it with appropriate interventions is unethical. Nevertheless, in support of the fact that the lack of blinding may not have significantly affected the study, the aim is that the influence of counseling targeted all participants and not selective to a group. In addition, a higher risk could have more intense counseling and possibly reduced the number of SAE. Fasting decisions were not much affected as only 7 out of the 93 high-risk patients did not fast compared to 5 out of 128 in the moderate-risk group. This is explained by the cohort’s firm intention to fast as Muslims and the shame and disappointment they feel by not fulfilling this major pillar of Islam. Therefore, they are counseled, but most of them fast as per this cohort; 96.4% did fast the whole month of Ramadan or attempted to do so. Patients intend to observe fasting during all Ramadan days and will only refrain from doing so in case of an illness to prevent a serious adverse effect.

Another area for improvement is the potential biases introduced through tele-interviews, with the risk of data collection being compromised by less rapport, probing, and interpretation of responses. Yet, tele-interviews contribute to better accessibility to participants, more effortless follow-up, and lower costs. In support of its use, evidence is lacking that they produce lower-quality data (20).

Finally, this study’s practical recommendation for clinicians is to incorporate evidence-based tools in risk assessment, such as the IDF-DAR risk assessment tool and FRAIL, into practice to aid in decision-making when assessing recommendations for added potential stress on patients, such as fasting.

## Conclusion

Fasting is not associated with a higher risk of adverse events, but the patient’s higher-risk category is. According to the IDF-DAR risk assessment, patients with diabetes in the low-risk category had a better outcome than those in the moderate- or high-risk categories regarding SAEs during Ramadan. Another independent risk factor is if the patient is frail, according to the FRAIL scoring. Future IDF-DAR risk assessment tool validation studies related to fasting need to control for social determinants of health and medication adjustment during fasting. Additionally, future research should aim at prospective and multi-regional validation to enhance the generalizability of the findings.

## Data Availability

The raw data supporting the conclusions of this article will be made available by the authors, without undue reservation.
